# News and Events of the Week

**Published:** 1910-01-01

**Authors:** 


### News and Events of the Week.

We have to announce the deaths of three members of one medical family within a fortnight: Mr. John James Rygate, M.B., Mrs. Rygate, and Mr. Reginald Rygate, L.R.C.P., M.R.C.S., on the 20th, 16th, and 6th of December respectively.

A meeting of the Essex Education Committee in London on December 20 decided that it was desirable that there should be systematic medical inspection of scholars attending secondary schools in the county. A special committee was appointed to report on details.

A new series of "National Health Manuals" is being prepared under the able editorship of Dr. T. N. Kelynack, whose name will be sufficient guarantee that the work will be of the best. All forms of social effort and enterprise, in so far as they touch medico-sociological problems, will be dealt with. Each chapter in every volume will be written by a well-known medical expert. The series, which will consist of twelve volumes, will be issued by Robert Culley. The first volume, dealing with "Infancy," will be issued early in the New Year.

"There are 264,000 feeble-minded persons in our country," said Mr. A. F. Tredgold, M.R.C.S., L.R.C.P., a member of the Royal Commission on the Care and Control of the Feeble-minded, who lectured upon that subject at Portsmouth last week. The meeting, which was held under the joint auspices of the Charity Organisation Society, the Citizens' Union, and the National Union of Women Workers, attracted a representative attendance of medical men, district nurses, and social workers of the town, and was presided over by the Rev. R. S. Medlicott, Vicar of Portsmouth.

In view of the General Election, the National Food Reform Association, of 178 St. Stephen's House, Westminster, S.W., has addressed to Parliamentary candidates a series of questions dealing with such subjects as the feeding of the Army and Navy, the inmates of prisons, workhouses, etc., underfed scholars, the milk supply, the teaching of cookery, etc., in schools, patent medicines, the publication by the Government of information as to the nutritive value of foodstuffs, and the treatment of inebriates. Copies may be obtained by sending a stamped addressed envelope to the secretary.

At an ordinary meeting of the Council of the Royal College of Surgeons on December 10, Mr. Henry T. Butlin presiding, the following recommendations of the Committee of Management of the Royal Colleges of Physicians and Surgeons were approved: "That the London School of Medicine for Women and the Edinburgh School of Medicine for Women be added to the list of recognised medical schools; that the Royal Free Hospital be added to the list of recognised hospitals; and that the Public Health Laboratories of the School of Medicine of the Royal Colleges of Physicians and Surgeons of Edinburgh be added to the list of laboratories recognised for the course of instruction for the diploma in Public Health."

The Annual Report of Livingstone College for the year 1908-9 is now being issued to subscribers. It is unfortunate that the missionary societies do not make better use of the College, and that the number of students was not so large during the session under review as in the previous session. If only the great British missionary societies would make systematic use of the College, the institution would soon be self-supporting. As it is, the financial year ended with a deficiency of £265, subscriptions and donations being £100 less than in the previous year and than the average for the last few years. This deficiency is partly due to the permanent improvements made in the College premises, but none the less it affords ground for generous support.

The Society of Tropical Medicine and Hygiene, at a meeting on December 17, unanimously passed the following resolutions: "That this society, recognising the great services rendered by Sir Alfred Jones, K.C.M.G., to tropical medicine, desires to record its appreciation of these services and sense of loss in his death. Further, that the secretaries be instructed to communicate this resolution to Sir Alfred Jones's family and to convey to them the sympathy of the society in their bereavement." "That the President be requested on behalf of the Fellows of this society to convey to Mr. Otto Beit their high appreciation of the great benefit which he has conferred on the study of disease, including tropical disease, by his munificent grant for the endowment of research."

For centuries medical knowledge in China has been at a standstill, and the population has been at the mercy of ignorant quacks who pass themselves off as doctors. Now, however, remarks *Mercy and Truth*, on all sides there is a rapidly growing demand for properly trained and qualified Chinese doctors. The establishment of well-equipped medical schools is imperative if the demand is to be supplied, and the missionary bodies working in China are naturally anxious to have their own schools. It is interesting to note that there are at present existing, or in prospect, sixteen medical schools which owe their inception to various missionary societies. The most active of these are, it is stated, the Union School, Pekin; the C.M.S. School at Hang-chow; and the School of St. John's University at Shanghai. The buildings of the C.M.S. Medical School at Fuh-chow are rapidly nearing completion, and will, it is hoped, be soon ready for occupation.

The Surrey Education Medical Officer, at a recent meeting, reported that the total number of children belonging to the specified age groups inspected during the past two months was 3,958. As a result of the inspections, 555 recommendations were made to parents to obtain necessary treatment for their children—*i.e.* 14.1 per cent. of the number inspected. The number of recommendations made for modification of school work of children requiring it was 237—that is, 5.9 per cent. of the number inspected. In addition to children belonging to the specified age groups, 1,073 children of other ages had been specially inspected on account of suspected defects, or for school attendance purposes. Special visits had also been paid to many schools for the purpose of examining children suspected to be verminous. The total number of exclusions from school notified as due, directly or indirectly, to all forms of communicable disease was 1,610. In the corresponding period of 1908 the total number of exclusions was 1,066.

